# P-1015. Cellular Composition of Bronchoalveolar Lavage Fluid in Patients with Hematologic Malignancies and Invasive Pulmonary Aspergillosis: Significant Associations with Peripheral Blood Cell Counts

**DOI:** 10.1093/ofid/ofae631.1205

**Published:** 2025-01-29

**Authors:** Sung-Yeon Cho, Sebastian Wurster, Takahiro Matsuo, Ajay Sheshadri, Dimitrios P Kontoyiannis

**Affiliations:** Division of Infectious Diseases, Department of Internal Medicine, College of Medicine, The Catholic University of Korea, Seoul, Korea, Seoul, Seoul-t'ukpyolsi, Republic of Korea; The University of Texas MD Anderson Cancer Center, Houston, Texas; The University of Texas MD Anderson Cancer Center, Houston, Texas; The University of Texas, MD Anderson Cancer Center, Houston, Texas; The University of Texas MD Anderson Cancer Center, Houston, Texas

## Abstract

**Background:**

Bronchoalveolar lavage (BAL) is frequently performed to diagnose invasive pulmonary aspergillosis (IPA) in patients (pts) with hematologic malignancies (HM). Given the critical role of the pulmonary immune environment for fungal control, BAL might be leveraged for diagnostic and prognostic immune biomarkers in IPA pts. However, data on the concordance of cell counts in peripheral blood (PB) and BAL fluid (BALF) of IPA pts is lacking.

**Figure 1.**
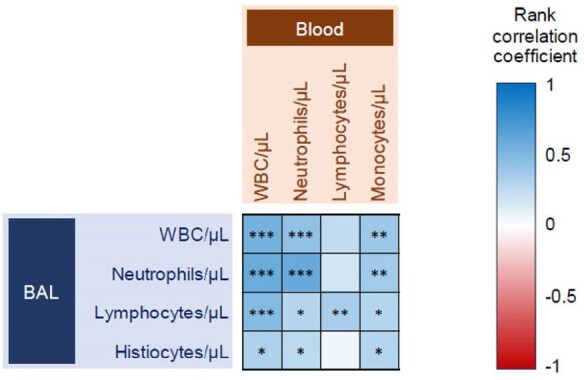
Heatmap summarizing Spearman’s rank correlation coefficients between peripheral blood white blood cell counts and differentials and those from bronchoalveolar lavage fluid at diagnosis of invasive pulmonary aspergillosis. * p<0.05, ** p<0.01, *** p<0.001.

**Methods:**

We retrospectively reviewed 63 culture-positive proven/probable IPA cases in adult pts who underwent bronchoscopy at MD Anderson Cancer Center in 2011–2022. BALF cell counts, microbiology data, PB white blood cell (WBC) differentials on the day of bronchoscopy, and IPA outcomes were analyzed.

**Figure 2.**
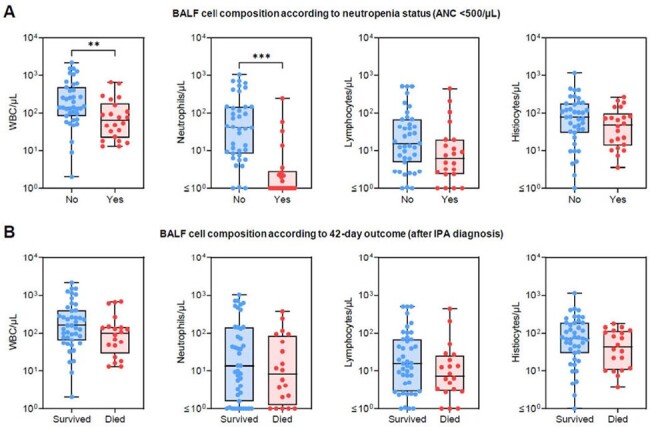
Comparison of bronchoalveolar lavage fluid cell composition according to neutropenia status (A) and 42-day outcomes (B) in invasive aspergillosis patients with hematologic malignancies. Mann-Whitney U test. ** p<0.01, *** p<0.001.

**Results:**

The median BALF WBC count at IPA diagnosis was 129/µL (interquartile range [IQR] 54–272/µL) and median neutrophil percentage was 11% (IQR 2–46%). PB total WBC counts significantly correlated with BALF WBC (*r*=0.55, *p*< 0.001), neutrophil (*r*=0.59, *p*< 0.001), and lymphocyte (*r*=0.48, *p*< 0.001) counts. Likewise, PB WBC differentials, i.e., absolute neutrophil and lymphocyte counts, significantly correlated with those in BALF (**Fig. 1**). Neutropenic pts (< 500/µL) had lower median BALF WBC (66 *vs*. 140/µL, *p*=0.005) and neutrophil counts (1 *vs*. 41/µL, *p*< 0.001) than non-neutropenic pts (**Fig. 2A**). BALF neutrophil counts were significantly higher in pts with bacterial coinfections than in those without coinfection (143 *vs*. 8/µL, *p*=0.005), while both neutrophil (37 *vs*. 5/µL, *p*=0.005) and lymphocyte (20 *vs*. 8/µL, *p*=0.021) counts were higher in pts with respiratory viral coinfection. Notably, cellular composition of BALF was not significantly associated with BALF galactomannan positivity or 42-day mortality after IPA diagnosis (**Fig. 2B**).

**Conclusion:**

Unlike the well-known association between PB cytopenias and IPA outcome, severity of IPA was not significantly influenced by quantitative BAL cell composition, underscoring the unmet need for reliable qualitative/functional BAL biomarkers to prognosticate fungal control in HM pts. Both PB cytopenias and coinfections significantly affected immune cell mobilization into BALF. Thus, clinical context will be critical for normalization and validation of BAL-based host biomarkers.

**Disclosures:**

**Sebastian Wurster, MD, MSc**, Astellas Pharma: Grant/Research Support|Gilead Sciences: Grant/Research Support **Dimitrios P. Kontoyiannis, MD**, AbbVie: Advisor/Consultant|Astellas Pharma: Advisor/Consultant|Astellas Pharma: Grant/Research Support|Astellas Pharma: Honoraria|Cidara Therapeutics: Advisor/Consultant|Gilead Sciences: Advisor/Consultant|Gilead Sciences: Grant/Research Support|Gilead Sciences: Honoraria|Knight: Advisor/Consultant|Merck: Advisor/Consultant|Scynexis: Advisor/Consultant

